# MiR206 and 423-3p Are Differently Modulated in Fast and Slow-Progressing Amyotrophic Lateral Sclerosis Patients

**DOI:** 10.1007/s12017-024-08773-6

**Published:** 2024-03-15

**Authors:** Antonio Musarò, Gabriella Dobrowolny, Chiara Cambieri, Laura Libonati, Federica Moret, Irene Casola, Gaia Laurenzi, Matteo Garibaldi, Maurizio Inghilleri, Marco Ceccanti

**Affiliations:** 1 DAHFMO-Unit of Histology and Medical Embryology, Sapienza University of Rome, Laboratory Affiliated to Istituto Pasteur Italia-Fondazione Cenci Bolognetti, Via A. Scarpa 14, 00161 Rome, Italy; 2 Department of Human Neurosciences, Rare Neuromuscular Diseases Centre, Sapienza University of Rome, Viale Dell’Università 30, 00185 Rome, Italy; 3 Department of Neurology, Neuromuscular Disease Centre, Mental Health and Sensory Organs (NESMOS), Sant’Andrea Hospital, Sapienza University of Rome, 00189 Rome, Italy

**Keywords:** Amyotrophic lateral sclerosis, miRNA, Prognosis

## Abstract

Amyotrophic lateral sclerosis (ALS) is a rare neuromuscular disease with a wide disease progression. Despite several efforts to develop efficient biomarkers, many concerns about the available ones still need to be addressed. MicroRNA (miR) are non-coding RNAs that can modulate molecular circuits and are involved in ALS pathogenic mechanisms. 22 fast and 23 slow-progressing-defined ALS patients were recruited. ALSFRS-R, strength, respiratory function, nerve conduction studies, and creatine kinase were evaluated at the baseline and after 6 months of follow-up. The mean monthly reduction of the previous variables (progression index – PI) was calculated. MiR206, 133a-3p, 151a-5p, 199a-5p, and 423-3p were dosed. The univariate analysis showed an independent reduction of miR206 and an increase of miR423-3p in patients with a slow slope of ALSFRS-R and weakness, respectively. MiR206 and 423-3p are differently modulated in fast and slow-progressing ALS patients, suggesting a role for microRNAs in prognosis and therapeutic target.

## Background

Amyotrophic lateral sclerosis (ALS) is a rare worldwide disease characterized by neurodegeneration of the upper and lower motor neurons, usually leading to death 3 years after diagnosis, with a 5-year survival rate of 20–25% and a 20-year survival rate of 5% (Rowland & Shneider, 2001). Despite a huge effort, many aspects of this disease are still unknown. There is a great unmet need for cost-effective, reliable, accurate, non-invasive, and reproducible biomarkers for ALS. No certain biomarkers for diagnosis and prognosis are available for clinical practice (Turner & Benatar, 2015; Wilkins et al., 2021). Unlike other neurodegenerative diseases, finding a reliable biomarker for ALS is difficult due to the fast progression and the lack of a pre-symptomatic model (such as the mild cognitive impairment for Alzheimer’s disease).

Among the purposed biomarkers for ALS, neurofilaments (Nf), existing in the high (NfH), middle (NfM), and low molecular weight (NfL), are a cytoskeletal structure specifically synthesized in neurons (Yuan et al., 2017); they are considered a surrogate marker of axonal injury, degeneration, and loss (Petzold, 2005). NfL increase as early as 12 months before the disease onset (Benatar et al., 2018) and their level correlate with survival (Lu et al., 2015), but not with the functional diagnostic El-Escorial scores (Feneberg et al., 2018). Even if a good correlation was found between cerebrospinal fluid (CSF) and plasma Nf (De Schaepdryver et al., 2018; Gille et al., 2019; Weydt et al., 2016), the diagnostic performance was found to be better in CSF compared to blood (De Schaepdryver et al., 2018; Li et al., 2016). Despite the good sensitivity, many concerns are arising about the specificity of Nf, mainly when tested in ALS-mimicking conditions. NfL are also purposed as a biomarker for neuropathies such as the familiar amyloidotic polyneuropathy (Maia et al., 2020; Romano et al., 2024), even if the best neuroanatomical correlation in ALS was demonstrated between upper motor neurodegeneration and the NfL (Gille et al., 2019; Poesen et al., 2017) and lower motorneuron degeneration and the plasmatic NfH (Poesen et al., 2017).

Other factors that have been considered as potential prognostic biomarkers that can differentiate fast and slow-progressing ALS patients are creatine kinase (CK) (Ceccanti et al., 2020; Rafiq et al., 2016; Tai et al., 2017), serum ferritin (Cheng et al., 2021), and creatinine (Guo et al., 2021). Among others, a surge of interest is observed in microRNAs (miRNAs). MiRNAs are well-conserved non-coding, single-strand short RNA sequences with a post-transcriptional role in the modulation of the gene expression, with a tissue-specific action (Quinlan et al., 2017; Saliminejad et al., 2019; Tx & Me, 2018). They can be found in tissues and the bloodstream, where they are associated with protein complexes (Argonaute protein family or lipoprotein) and encapsulated in vesicles (microparticles, exosomes, or apoptotic bodies), which protect them from the RNAases degradation. They bind the complementary messenger-RNA (mRNA) to form the RNA-induced silencing complex, which reduces or silences the RNA translation (Filipowicz et al., 2005; Quinlan et al., 2017). Recent studies report the identification of potential circulating miRNAs, namely miR4649-5p, miR424, miR133a, and miR206, as putative serum biomarkers for ALS pathogenesis (de Andrade et al., 2016; Kovanda et al., 2018; Takahashi et al., 2015). Other miRNAs, namely miR206, 133a-3p, 151a-5p, 199a-5p, and 423-3p, were modulated in ALS patients during a 30-month follow-up period (Dobrowolny et al., 2021). These last miRNAs were taken in account for this study, given the good relationship demonstrated with disease progression.

Nevertheless, beyond using different molecules as a biomarker for early disease onset and diagnosis detection, clinical practice needs reliable biomarkers for prognosis. The time of survival and the slope of the functional scores appear to be very different among the patients, according to many factors which could modulate the disease progression, such as the age of disease onset, the phenotype (i.e., spinal or bulbar) (Chiò et al., 2009), the time from disease onset to diagnosis, the gender and the functional scores at the first evaluation. A progression biomarker could be useful to select comparable groups in clinical trials too.

Moreover, different endpoints can be considered for prognosis, such as the time of survival or the time to tracheostomy/PEG. A well-characterized prognostic endpoint is the ALSFRS-R slope (progression index) (Benatar et al., 2018; Elia et al., 2016; Shefner et al., 2020), representing the average monthly reduction of the Revised ALS functional rating scale over time. A similar approach can be used for the prognosis of respiratory, strength, and neurophysiologic testing, using the forced vital capacity (FVC), the medical research council (MRC) score for strength, and the compound muscle action potential (cMAP) slope in the nerve conduction studies.

This study aimed to evaluate the relationship of selected miRNAs with the clinical and neurophysiologic scores and their role as dependent or independent predictors of disease progression in a consistent cohort of ALS patients.

## Methods

A sample of forty-five-defined ALS patients (revised El-Escorial criteria) was selected for this retrospective observational study from a cohort of 159 ALS consecutive patients enrolled at the referral center for rare neuromuscular diseases of Sapienza University of Rome. The sample, divided into 22 fast and 23 slow progressors according to the 6-month after admittance ALSFRS-R slope (cut-off 0.5 points/month with 0.5 considered slow progressor), was balanced in terms of baseline ALSFRS-R, age, and phenotype (spinal or bulbar onset). All the selected patients underwent serum storage at the first ambulatory access and followed up for 6 months. At the baseline and the 6-month follow-up visit, the time from disease onset (expressed in months), ALSFRS-R score, MRC score from the upper and lower limbs, cMAP from right medial plantar and left ulnar nerves, forced vital capacity (FVC), and the serum creatine kinase (CK) were registered. A slope of progression (average points/month loss) was calculated for all the previous variables. Expert neurologists for ALS diagnosis performed all the clinical scales and tests.

### MiRNAs Extraction and Real-Time Analysis

Circulating microRNAs were extracted from 200 µl of serum by miRNeasy serum/plasma kit (Qiagen Cat. 217184). During the extraction, 1.43 µl of Spike-In kit (Qiagen Cat. 339390) was added to each sample to allow the relative quantification of microRNAs.

Subsequently, 10 µl of the isolated microRNAs were retrotranscribed by miRCURY® LNA® RT Kit (Cat. 339340), and cDNA was diluted 1:10. Finally, 4 µl of cDNA were used for quantitative Real-Time PCR through QuantStudio™ 7 Flex Real-Time PCR System (Thermo Fisher Scientific), using miRCURY LNA miRNA Probe PCR Assay. To quantify the levels of the analyzed circulating microRNAs, the Quantitative RT-PCR sample value was normalized for the expression of the Unisp2 Spike-In template. The relative level for each miRNA was calculated using the 2^− ΔΔCt^ method (Livak & Schmittgen, 2001) and reported as fold change.

### Statistical Analysis

Demographic data were expressed as the average ± standard error mean (SEM). The independent sample T-test was used to assess the levels of the single miRNAs between fast and slow progressors once the variance was demonstrated homogeneous in the two groups. Linear (Pearson coefficient) and curve estimation models were used for regression analysis. ROC curve was used to calculate the best cut-off value of miRNAs to discriminate fast and slow progressor ALS patients, once a between-groups difference was demonstrated. General linear model (GLM) univariate analysis was used to test regression analysis and analysis of variance for one dependent variable by one or more factors and/or variables. IBM SPSS version 27.0 was used for the data analysis; statistical significance was set as *P* < 0.05.

## Results

Fast and slow-progressing ALS patients were well-balanced in terms of age, gender, ALSFRS-R score, onset phenotype, total MRC, MRC subscores for upper and lower limbs, FVC, neurophysiological scores, and BMI at the baseline (Table 1). Table 2 shows differences of the progression indexes calculated for clinical and instrumental scores.

**Table 1:** Demographic data

	Fast	Slow	*P* value
N	22	23	
Age	62.5 ± 2.25	64.1 ± 2.2	> 0.05
Sex: M/F	10/12	12/11	> 0.05
Onset phenotype: spinal/bulbar	16/6	18/5	> 0.05
Baseline ALSFRS-R	37.27 ± 0.79	36.61 ± 0.87	> 0.05
ALSFRS-R progression index	− **1.02 ± 0.15**	− **0.23 ± 0.03**	** < 0.001**
Total MRC	126.5 ± 3.31	124.0 ± 4.09	> 0.05
MRC upper limbs	68.0 ± 1.72	66.87 ± 2.44	> 0.05
MRC lower limbs	58.5 ± 3.1	57.13 ± 4.16	> 0.05
FVC	73.14 ± 4.75	77.74 ± 3.42	> 0.05
Medial plantar cMAP amplitude	8.49 ± 1.59	8.67 ± 1.73	> 0.05
Ulnar cMAP amplitude	8.45 ± 1.02	8.29 ± 0.97	> 0.05
BMI	23.82 ± 1.52	24.91 ± 0.92	> 0.05

Bold values indicate statistically significant

Results are expressed as mean ± SEM

*ALSFRS-R* Revised ALS functional rating scale, *MRC* medical research council scale for strength, *FVC* forced vital capacity (expressed as percentage of normal values), *cMAP* compound muscle action potential (expressed in mV), *BMI* body mass index

**Table 2:** Demographic data

	Fast	Slow	*P* value
N	22	23	
PI ALSFRS-R Tot	**− 0.76 ± 0.19**	**− 0.52 ± 0.09**	** < 0.01**
PI ALSFRS-R S	**− 0.48 ± 0.1**	**− 0.35 ± 0.07**	** < 0.01**
PI ALSFRS-R B	− 0.22 ± 0.1	− 0.09 ± 0.03	= 0.08
PI ALSFRS-R R	**− 0.11 ± 0.07**	**− 0.09 ± 0.04**	** < 0.05**
PI MRC Tot	**− 2.38 ± 0.5**	**− 1.09 ± 0.24**	** < 0.01**
PI medial plantar cmap	− 0.18 ± 0.19	− 0.16 ± 0.1	> 0.05
PI ulnar cMAP	**− 0.31 ± 0.09**	**− 0.24 ± 0.09**	** < 0.05**
PI FVC	− 3.76 ± 1.98	− 1.66 ± 0.66	> 0.05

Bold values indicate statistically significant

Results are expressed as mean ± SEM

*PI* progression index, calculated as the (score at T1–T0)/months, *ALSFRS-R* Revised ALS functional rating scale, *MRC* medical research council scale for strength, *FVC* forced vital capacity (expressed as percentage of normal values), *cMAP* compound muscle action potential (expressed in mV), *BMI* body mass index

### Differences in miRNA Levels Between Fast and Slow-Progressing ALS Patients

**Fig. 1:**
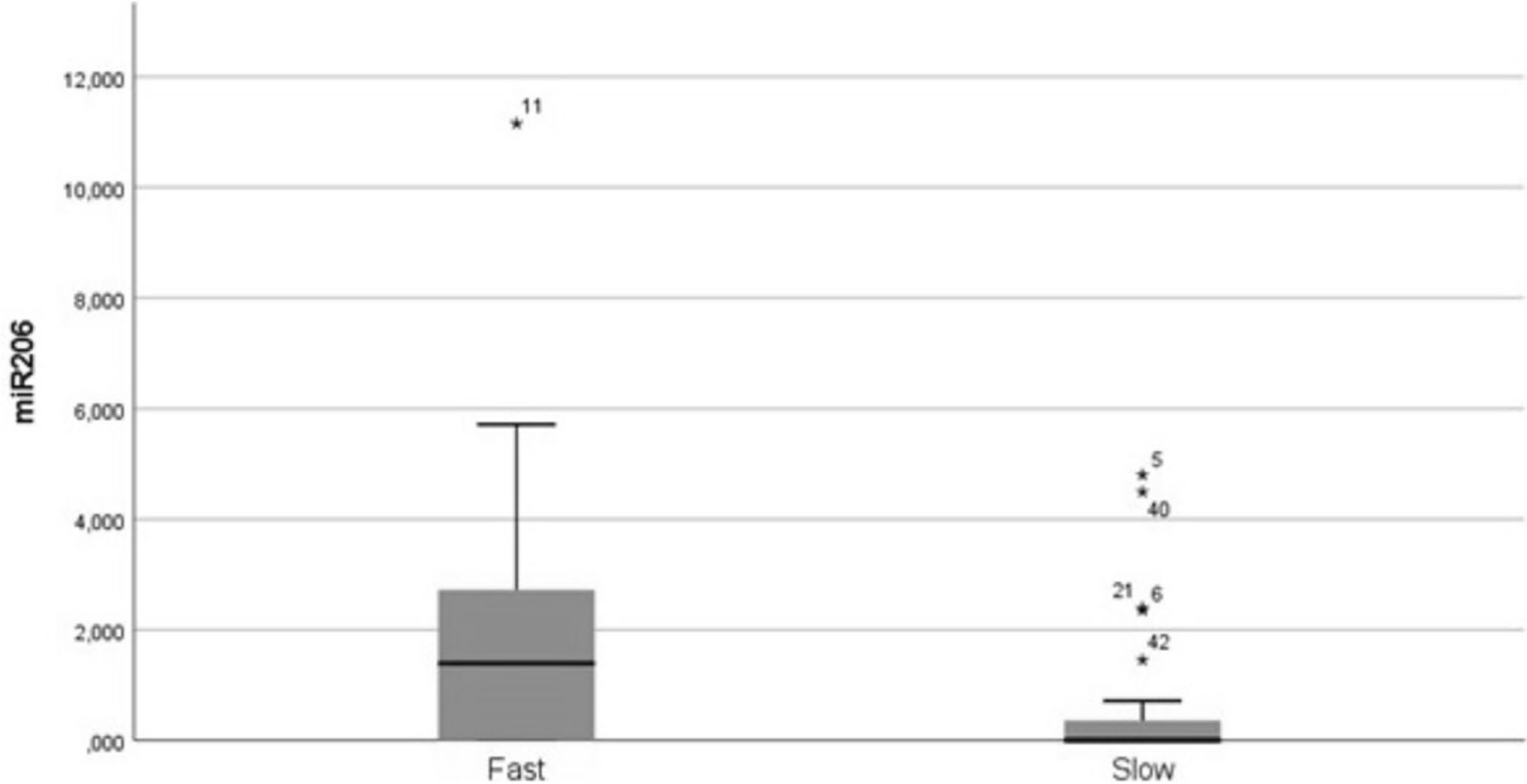
Mir206 in fast and slow ALS progressors

No different levels of miR133a-3p, 151a-5p, 199a-5p, and 423-3p were detected between the two groups. Mir206 was significantly higher in the fast vs slow-progressing group (283 higher mean – Tab. 3 and Fig. 1), with 7 slow progressors showing no detectable levels of miR206; a cut-off value of 0.245-fold change showed 63.6% of sensitivity and 73.9% of specificity to discriminate fast and slow progressors. MiRNAs’ relative dosages are reported in Table 3.

**Table 3:** MiR relative dosage

	Fast	Slow	*P* value
MiR133a-3p	0.791 (± 0.145)	1.828 (± 1.066)	> 0.05
MiR151a-5p	0.757 (± 0.091)	0.696 (± 0.089)	> 0.05
MiR199a-5p	2.225 (± 0.362)	2.528 (± 0.458)	> 0.05
MiR206	**1.994 (± 0.573)**	**0.704 (± 0.300)**	** < 0.05**
MiR423-3p	1.023 (± 0.138)	1.305 (± 0.193)	> 0.05

Bold values indicate statistically significant

Results are expressed as fold change ± SEM

### Regression Analysis

**Fig. 2:**
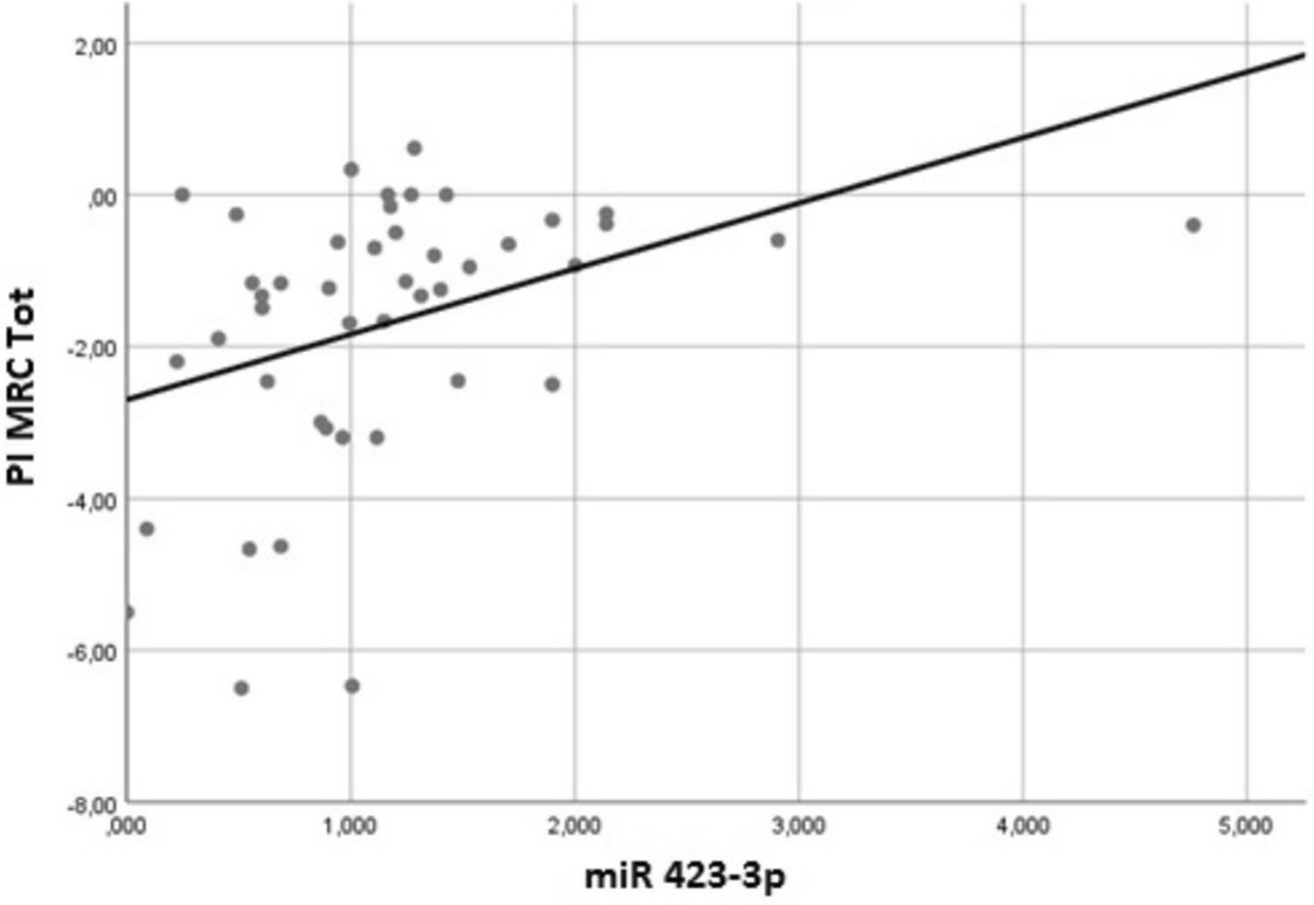
Linear correlation between progression index of the total MRC and miR423-3p

The single miRNAs were correlated to all the clinical and neurophysiological scores and the calculated progression of slopes. MiR199a-5p was directly related to CK values (*P* = 0.011); miR423-3p was inversely related to the baseline total MRC score (upper + lower limbs- *P* = 0.011-); miR423-3p was also strictly related to the slope of total MRC (*P* = 0.007), with higher values related to slow MRC progression (Fig. 2). Time from the disease onset to diagnosis was related to the progression index of the ALSFRS-R (linear correlation—*P* < 0.001) and MRC (logarithmic relationship—*P* = 0.024), with patients with a long-lasting disease associated with a better prognosis.

### GLM Univariate Analysis

One-way ANCOVA was conducted to determine a statistically significant difference between fast and slow progressors on miR423-3p controlling for months from the disease onset, baseline ALSFRS-R score, baseline total MRC score, and MRC slope. The overall model was significant (*P* = 0.003, eta^2^ = 0.377); higher levels of miR423-3p were associated with higher MRC progression index as an independent variable, i.e., better prognosis (Beta coefficient: 0.220, 95%CI 0.073 to 0.366; adjusted *P* = 0.004).

The same analysis was run with fast vs slow progression as the dependent variable, controlled for age, time from disease onset and miR206 as a covariate. The overall model was significant (*P* < 0.001, eta^2^ = 0.379); higher level of miR206 and low months from disease onset were independent predictors of progression rate (Beta coefficient: − 0.072 and 0.011 respectively, 95%CI 0.132 to − 0.012 and 0.005 to 0.016, respectively; adjusted *P* = 0.021 and 0.001, respectively), with high miR206 levels and few months from disease onset to diagnosis associated to fast progression rate.

## Discussion

ALS is a neurodegenerative disease with a poor prognosis. Different groups of patients are clinically identifiable in terms of the progression rate of ALSFRS-R score, MRC, and FVC deterioration. Even if the differences are evident in the aftermath, no biomarker has been validated to predict the prognosis in a single patient. In this paper, we propose the microRNA as biomarkers to predict fast or slow disease progression, according to the progression index of ALSFRS-R, MRC, and FVC. A strength of this study is the clinical uniformity between the fast and slow patient group, as highlighted by the demographic data shown in Table 1.

MiR206 was significantly higher in fast compared to slow-progressing ALS. A cut-off value of 0.245-fold change showed acceptable sensitivity and specificity. A long time from disease onset to diagnosis was associated with a slow progression rate in terms of ALSFRS-R and MRC score, probably mirroring the difficulties in diagnosing ALS in patients with slow symptoms progression and a lower impact of the disease on the patient’s life. High levels of miR206 and a few months from disease onset were associated with fast progression in ALSFRS-R even when adjusted for the age at the time of diagnosis.

High levels of miR423-3p were independently associated with a slow MRC progression even when normalized for baseline ALSFRS-R and MRC. Given the inverse relationship between miR423-3p and baseline MRC, a ceiling effect regarding the MRC PI could prospect patients with high miR423-3p levels and low baseline MRC scores. The independent role of MRC PI on miR423-3p was confirmed even when adjusted for the baseline MRC, thus, excluding this possibility and confirming the independent prognostic value.

These data outline a model where ALS patients with high miR423-3p and low miR206 are slowly progressive.

MiR206 is a regulatory factor induced by denervation in ALS and exclusively expressed in the muscle; its role is to prevent the deleterious action of histone deacetylase 4 (HDAC4) on reinnervation. HDAC4 also induces myogenic expression by repressing Dach2 expression, a repressor of myogenin (Cohen et al., 2007; Tang et al., 2009). Mutant miR206^−/−^ ALS mice show a fast progression and short survival, increasing myogenin transcript (Williams et al., 2009). Interestingly, miR206 is highly expressed in slow-twitch muscles which are the most resistant to denervation in ALS models (Pun et al., 2006). In this context, the increased level of miR206 in the fast-progressing ALS patients suggests a muscle-activated compensatory mechanism, which unsuccessfully promotes nerve–muscle interaction in the highly denervated fast-progressing ALS muscle. Other papers described a modulation of miR206 in ALS model, with different results. Toivonen et al. demonstrated increased levels of miR206 in a mouse ALS model and a small sample of ALS patients (2014), suggesting to use this muscle-specific microRNA as a biomarker and highlighting the role of muscle in ALS. Another recent paper demonstrated high levels of miR206 associated to slow-progressing ALS (Dobrowolny et al., 2021); in this paper, just 11 slow and 6 fast-progressing ALS patients were analyzed. Moreover, different methods were used for miRNA dosage and for the definition of progression rate, with fast and slow progressors defined according to relative ALSFRS-R progression within the cohort instead of absolute once. All these reasons impair a comparison between the two studies.

MiR423-3p was found upregulated in slow compared to fast-progressing ALS patients (related to MRC PI), once controlled for months from the disease onset, ALSFRS-R score, total MRC score. MiR423-3p was previously demonstrated to inhibit Bim, an important pro-apoptotic protein in cancers (X. Li et al., 2021), which has been demonstrated to be down-modulated in a slow-progressing ALS mouse model (Matus et al., 2013). Bim silencing also decreased Bax recruitment to mitochondria and cytochrome c redistribution in a SOD1 murine neuroblastoma cell line (Soo et al., 2012). Thus, a possible protective role for miR423-3p could be exerted inhibiting the Bim-mediated motor neuron apoptosis.

## Conclusions

All these data demonstrate a possible value of specific miRNAs as prognostic factors in ALS, suggesting some cues for pathogenic and adaptive mechanisms and possible molecular targets. The role of miR206, a typical muscle-specific miRNA, confirms the role of muscle in modulating the disease progression in ALS.

## Data Availability

The datasets used and/or analyzed during the current study are available from the corresponding author on reasonable request.

## Abbreviations

ALS: Amyotrophic lateral sclerosis
ALSFRS-R: Revised ALS functional rating scale
BMI: Body mass index
CK: Creatine kinase
cMAP: Compound muscle action potential
CSF: Cerebrospinal fluid
FVC: Forced vital capacity
GLM: General linear model
MiR: MicroRNA
MRC: Medical research council
mRNA: Messenger-RNA
Nf: Neurofilaments
NfL: Light molecular weight neurofilaments
NfM: Medium molecular weight
NfH: High molecular weight neurofilaments
OSAS: Obstructive sleep apnea syndrome
PI: Progression index
SEM: Standard error mean
